# Fitness Outcomes Related to Glyphosate Resistance in Kochia (*Kochia scoparia*): What Life History Stage to Examine?

**DOI:** 10.3389/fpls.2017.01090

**Published:** 2017-06-30

**Authors:** Omobolanle Adewale Osipitan, Johanna Anita Dille

**Affiliations:** Department of Agronomy, Kansas State UniversityManhattan, KS, United States

**Keywords:** kochia, ecological fitness, glyphosate resistant, seed germination, vegetative growth and fecundity

## Abstract

A fast-spreading weed, kochia (*Kochia scoparia*), has developed resistance to the widely-used herbicide, glyphosate. Understanding the relationship between the occurrence of glyphosate resistance caused by multiple EPSPS gene copies and kochia fitness may suggest a more effective way of controlling kochia. A study was conducted to assess fitness cost of glyphosate resistance compared to susceptibility in kochia populations at different life history stages, that is rate of seed germination, increase in plant height, days to flowering, biomass accumulation at maturity, and fecundity. Six kochia populations from Scott, Finney, Thomas, Phillips, Wallace, and Wichita counties in western Kansas were characterized for resistance to field-use rate of glyphosate and with an *in vivo* shikimate accumulation assay. Seed germination was determined in growth chambers at three constant temperatures (5, 10, and 15 C) while vegetative growth and fecundity responses were evaluated in a field study using a target-neighborhood competition design in 2014 and 2015. One target plant from each of the six kochia populations was surrounded by neighboring kochia densities equivalent to 10 (low), 35 (moderate), or 70 (high) kochia plants m^−2^. In 2015, neighboring corn densities equivalent to 10 and 35 plants m^−2^ were also evaluated. Treatments were arranged in a randomized complete block design with at least 7 replications. Three kochia populations were classified as glyphosate-resistant (GR) [Scott (SC-R), Finney (FN-R), and Thomas (TH-R)] and three populations were classified as glyphosate-susceptible (GS) [Phillips (PH-S), Wallace (WA-S) and Wichita (WI-S)]. Of the life history stages measured, fitness differences between the GR and GS kochia populations were consistently found in their germination characteristics. The GR kochia showed reduced seed longevity, slower germination rate, and less total germination than the GS kochia. In the field, increases in plant height, biomass accumulation, and fecundity were not clearly different between GR and GS kochia populations (irrespective of neighbor density). Hence, weed management plans should integrate practices that take advantage of the relatively poor germination characteristics of GR kochia. This study suggests that evaluating plant fitness at different life history stages can increase the potential of detecting fitness costs.

## Introduction

Differences in the relative fitness of herbicide-resistant (HR) and -susceptible (HS) weed genotypes influences the dynamics of mixed HR and HS populations by shifting their proportions over time (Gressel and Segel, 1982). Under field conditions, HR and HS plants interact with each other and with other plant species. The biotype with greater fitness is expected to out-compete the relatively less fit biotype. Since many mutations in plants have deleterious, pleiotropic effects on their likelihood of survival and/or reproduction (Roles and Conner, 2008), it is generally assumed that herbicide resistance mutations will be associated with an initial cost to a plant's fitness (Vila-Aiub et al., 2015). The resource-based allocation theory suggests that there is a trade-off between allocation of resources for plant growth and for plant defense (herbicide resistance) against environmental stress (Herms and Mattson, 1992; Gassmann, 2005).

The assumption that HR plants are less fit than HS plants in the absence of herbicide application is mostly based on early studies of triazine resistance (Vila-Aiub et al., 2015). These studies indicated a marked reduction in the vegetative and reproductive success of triazine-resistant biotypes relative to susceptible biotypes when triazine herbicides were not applied (as reviewed by Jasieniuk et al., 1996). The most common mechanism for triazine resistance, mutation in the quinone B binding domain of D1 protein in PS II, decreases the binding affinity for triazine molecules to the protein (Devine and Shukla, 2000). This mutation reduces efficiency of PS II, which results in reduced plant fitness. Although, fitness cost has been widely detected in triazine-resistant plants, this cannot be generalized for resistance to other herbicide modes of action, such as glyphosate, a 5-enolpyruvylshikimate 3-phosphate synthase (EPSPS) inhibitor. A genetic mechanism responsible for glyphosate resistance in kochia has been reported to be amplification of EPSPS gene which translates to overproduction of EPSPS enzyme in plant (Jugulam et al., 2014; Godar et al., 2015), and it is expected that more metabolic energy will be required for enzyme production at the detriment of other plant biological functions or developmental processes compared to normal EPSPS production in a susceptible individual.

Efforts are still being made to identify how evolution of glyphosate resistance in weeds can result in differential fitness, and reports so far have shown that fitness differences between GR and GS cannot be generalized across plant species (Vila-Aiub et al., 2014; Wang et al., 2014; Kumar and Jha, 2015). Reports also suggest that GR plants' fitness varied by mechanism of glyphosate resistance (Preston and Wakelin, 2008; Preston et al., 2009), genetic background of weed species (Giacomini et al., 2014), and environmental stress, such as competition for resources (Davis et al., 2009; Shrestha et al., 2010).

Accurate quantitative estimates of the relative fitness of resistant and susceptible plants in the absence of herbicide have been difficult to obtain (Jasieniuk et al., 1996; van Etten et al., 2016). A more appropriate comparison to estimate differential fitness of resistant and susceptible plants is to compare plants within the same weed population (Pedersen et al., 2007) or use of isogenic lines (Vila-Aiub et al., 2015) to reduce the effect of genetic background on fitness analysis. However, studies have generally compared the fitness of resistant and susceptible plants from very different and geographically separate populations (Jacobs et al., 1988; Stowe and Holt, 1988; Holt, 1990; Thompson et al., 1994; Shrestha et al., 2010; van Etten et al., 2016), and of these, some compared any resistant and only one susceptible population (Mortimer et al., 1992; Marshall et al., 1994; Shrestha et al., 2010). To control confounding effects of plant response to resistance or susceptible traits and genetic background resulting from environmental experience, care must be taken to select populations from the same geographic area or growing conditions (Jasieniuk et al., 1996), and several resistant and susceptible populations should be compared (Cousens et al., 1997; Strauss et al., 2002; van Etten et al., 2016).

Kochia produces protogynous flowers where the stigmas emerge before anther development and this necessitates outcrossing. Thus, cross pollination within and among plants is common in kochia. Repeated use of glyphosate and outcrossing among populations would potentially promote development of glyphosate resistance in kochia populations. Maintaining homogeneity in kochia through inbreeding over generations has been difficult perhaps due to inbreeding depression as observations showed that such seeds either have low viability or improperly formed (Esser, 2014). In this study, multiple kochia populations were collected from fields in an irrigated crop rotation of grain sorghum and glyphosate-resistant (GR) soybean in western Kansas. These populations were compared in a common garden to check for differences in the original environment of these populations. A qPCR study showed that individuals in our GR populations had higher EPSPS gene copy number (the mechanism responsible for glyphosate resistance) while the GS populations had normal EPSPS gene copy numbers (Osipitan, 2016).

Measuring fitness throughout a plant's life cycle has also been recommended (Vila-Aiub et al., 2015; van Etten et al., 2016). Examining a single life history stage may not be sufficient to identify differential fitness for resistance traits (Pedersen et al., 2007; Lamego et al., 2011; Lehnhoff et al., 2013). Subjecting each of these life history stages to either abiotic or biotic environmental stress has helped identify fitness differences between HR and HS weed biotypes. For example, the influence of temperature on rate of weed seed germination has been widely explored to explain the differences between HR and HS weed biotypes (Thompson et al., 1994; Park et al., 2004; Vila-Aiub et al., 2005; Elahifard and Mijani, 2014; Tang et al., 2015). Many research studies have also used densities or crowding to subject plant biotypes to competition in order to measure their fitness through vegetative and reproductive responses (Légère et al., 2000; Vila-Aiub et al., 2005; Shrestha et al., 2010; Travlos and Chachalis, 2013). Two main competition designs used to evaluate differential growth and/or fecundity of HR and HS weed populations include target-neighborhood design (Vila-Aiub et al., 2005, 2009a) and replacement series (Légère et al., 2000; Shrestha et al., 2010; Travlos and Chachalis, 2013). The advantage of target-neighborhood over replacement series design is the ability of the former to allow the study of competition effect or response under varying densities with two or more biotypes being compared.

The objectives of this study were to (1) characterize six kochia populations from western Kansas into glyphosate-resistant (GR) and glyphosate-susceptible (GS) populations and (2) evaluate fitness costs as a result of evolution of glyphosate resistance in these kochia populations at three life history stages: seed germination, plant vegetative growth, and fecundity.

## Materials and methods

Six kochia populations were collected from different fields in western Kansas. Kochia seeds were collected from one suspected GR population in Thomas County in 2007 and seeds from five other populations were collected in 2012, including suspected GR populations in Scott and Finney Counties and suspected GS populations from Phillips, Wallace and Wichita Counties. Seeds were harvested from more than 10 mature plants per site and bulked in separate bags for each population. Seeds were stripped off plants and cleaned using an air column separator. Efforts were made to ensure that seeds from the 10 plants for each population were equally bulked. To minimize loss of seed quality, the collected seeds were stored in cold (≤ −5 C).

### Characterization of populations to GR and GS: glyphosate discriminatory dose assay

Seeds of each kochia population were sown in 28 by 6 by 8 cm flats filled with moisture control potting mix (Miracle Gro, Marysville, OH) and grown in the Department of Agronomy—Weed Science greenhouse at Kansas State University, Manhattan, KS. The greenhouse was maintained at 25/20 C day/night, photoperiod of 15/9 h day/night enhanced with 120 μmol m^−2^ s^−1^ illumination provided by sodium vapor lamps, and about 60% relative humidity. Once 2–3 cm tall, individual seedlings were transplanted into 8.5 by 8.5 by 7 cm plastic pots filled with the same potting mix. Glyphosate (Roundup WeatherMax, Monsanto Company, St. Louis, MO) at a field use rate (1X, 0.84 kg ae ha^−1^) and double the field use rate (2X, 1.68 kg ae ha^−1^) along with 2% v/v liquid ammonium sulfate were applied to individual plants when they reached 8–10 cm in height. Glyphosate was applied in a cabinet spray chamber (Research Track Sprayer, De Vries Manufacturing, P.O. Box 184, Hollandale, MN). The spray chamber had a flat-fan nozzle tip (80015LP TeeJet tip, Spraying Systems Co., P.O. Box 7900, Wheaton, IL) that delivered a spray volume of 168 L ha^−1^ using a pressure of 220 kpa with a speed of 4.8 km h^−1^. Glyphosate treatments were replicated 10 and 7 times for first and second runs, respectively. There were also five untreated plants for each population as controls in each run. The study was laid out in a completely randomized design on experimental benches in the greenhouse.

Plant survival and injury score were recorded at 4 week after glyphosate treatment. Survival was measured as percentage of living plants compared to total plants sprayed. Plant injury score was on the scale of 1–9, where 1 represented no injury and 9 represented completely injured or dead plants. Injury score response of populations to glyphosate treatment were tested with a one-way ANOVA in R version 3.2.3 (R Core Team, 2015) and Tukey's Honestly Significant Difference (α = 5%) was used to compare injury score means among populations.

### Characterization of populations to GR and GS: shikimate accumulation assay

The six kochia populations were assessed for shikimate accumulation to further validate the characterization of GR and GS populations. In plants, glyphosate inhibits production of the aromatic amino acids (tryptophan, phenylalanine and tyrosine) in the shikimic acid pathway causing a build-up of shikimate-3-phosphate, a substrate of EPSPS and its dephosphorylated state, shikimate (Shaner et al., 2005). Both GR and GS plants are expected to accumulate shikimate after exposure to glyphosate but the levels are much higher in GS plants (Wiersma et al., 2015). A measure of shikimate accumulation was determined following the procedure developed by Shaner et al. (2005) and modified by Godar (2014). Plants of each population were grown in the greenhouse in a similar method and condition described above. Ten representative plants that were 10 cm tall were selected from each population. Four 5-mm leaf disks were collected from two fully-expanded young leaves from each plant. Shikimate accumulation in plants from the six kochia populations were analyzed using one-way ANOVA to test if there was difference among populations and the means were compared using Tukey's Honestly Significant Difference (α = 5%).

### Germination study

Seeds were stored in cold (≤ −5 C) until germination study was conducted in 2015. The TH-R kochia population was not included in the germination study because of seed age difference (collected in 2007) compared with other kochia populations which were of the same age (all collected in 2012). Germination rates of the five populations were evaluated at three constant temperatures of 5, 10, and 15 C in darkened growth chambers. A conducive temperature for kochia germination was 10 C (Everitt et al., 1983). Three petri-dishes (100 by 15 mm) of 30 seeds for each of the five populations were placed into one of the three growth chambers set at 5, 10, or 15 C. Dishes within a chamber were arranged in a completely randomized design with three replications. The dishes contained one filter paper (Whatman No 2) soaked with 3 ml of distilled water and moistened with additional water as needed throughout the study. The temperature in each growth chamber was constantly monitored with an internal thermometer. A seed was considered germinated when the radicle was about 2 mm long. Germination counts were done every 12 h for the first 144 h (6 d) and subsequent germination counts were done every 24 h for a total of 21 d. Germinated seeds were removed after each count. The initial 12 h observation interval was to capture the lag and rapid germination stages, while the 24 h observation interval would capture remaining germination. After the final germination count, un-germinated seeds in the petri dishes were re-wetted 7 days later. The viability of the re-wetted seeds was tested through finger press for hardness after 78 h at 24 C (a modified procedure of Davis et al., 2005). Relatively hard seeds were considered viable while easily ruptured seeds were considered unviable. Both germinated seeds and hard seeds after wetting accounted for the total viable seeds.

A non-linear three parameter logistic regression model was used to describe germination dynamics fit in R version 3.2.3 (R Core Team, 2015):

(1) $$\begin{array}{l} \text{y}=\text{d}/[1+{\left(\text{t}/{\text{t}}_{\text{o}}\right)}^{\text{b}}] \end{array}$$

where y is cumulative germination (%) at time t (h), d is maximum cumulative germination, t_o_ is time (h) required to reach 50% of maximum cumulative germination, and b is slope of function around t_o_. Analysis of variance was conducted to determine the effect of temperature on parameter estimates among populations and parameter estimates were ranked based on Tukey's Honestly Significant Difference (α = 5%) in R V 3.2.3 (R Core Team, 2015).

### Vegetative growth and fecundity study

The vegetative and seed production fitness of the six kochia populations were evaluated in a field experiment which was very different from greenhouse or partial field studies (where plants were placed in pots in an open field) used in most ecological fitness experiments (Vila-Aiub et al., 2009a; Shrestha et al., 2010; Wang et al., 2014; Kumar and Jha, 2015). The field experiment was conducted at the Kansas State University Department of Agronomy Ashland Bottoms Experiment Field (39.12577 N 96.6365 W) near Manhattan, KS in 2014 and 2015. The two locations were within 1 km of each other. The soil series of the field was a Wymore, which is a moderately well drained silty clay loam soil formed in loess (Natural Resources Conservation Service - US Department of Agriculture, 2016). In 2014, results of a soil analysis were pH (7.1), nitrogen (17 ppm), phosphorus (26.5 ppm), potassium (339 ppm), organic matter (2.5%), and cation exchange capacity (13.1 meq 100 g^−1^). In 2015, the soil analysis results were pH (6.2), nitrogen (78.9 ppm), phosphorus (47.1 ppm), potassium (529 ppm), organic matter (2.5%), and cation exchange capacity (13.1 meq 100 g^−1^).

**Figure 1:**
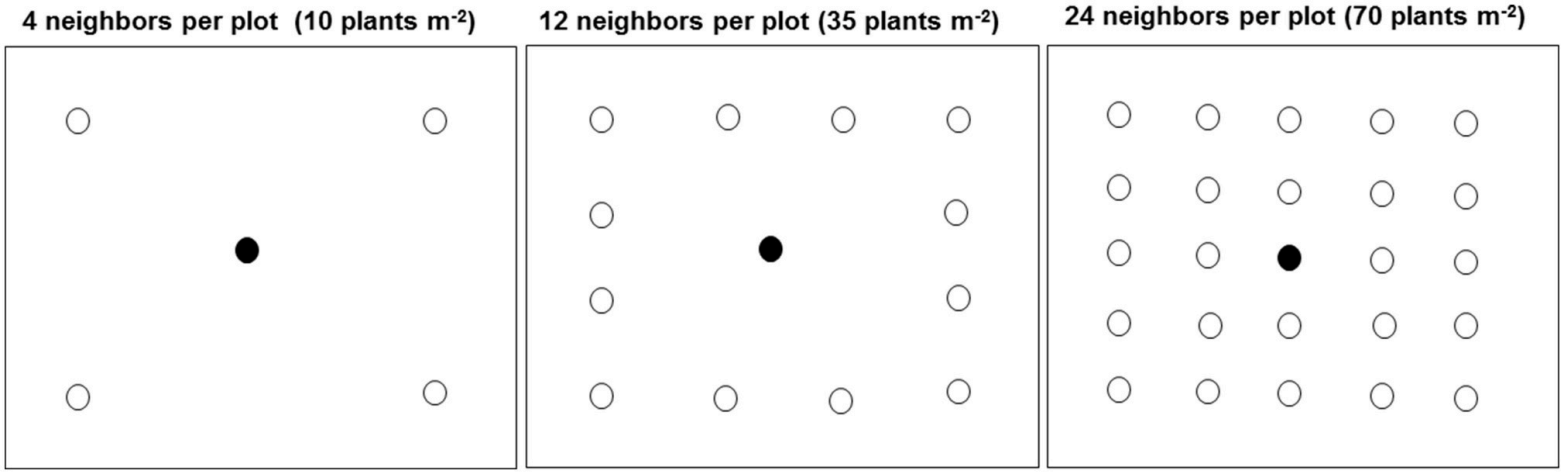
Distribution of target and neighbor kochia plants at different densities in each plot (0.6 by 0.6 m) in the field. The closed and open circles were the target and neighboring plants respectively. For the 10 and 35 plants m^−2^, the target to neighbor distant was about 15 cm while for the 70 plants m^−2^, the distance from target to the immediate neighbor and between neighbors was about 10 cm.

The density treatment design was a modified form of target-neighborhood method used by Vila-Aiub et al. (2009a). The distribution of target-neighbor plants was illustrated in Figure 1. In 2014, one target plant from each of the six kochia populations was surrounded by its own population of neighboring kochia at densities equivalent to 10 (low), 35 (moderate), or 70 (high) plants m^−2^. The two treatment factors were six kochia populations and three neighbor densities. Treatments were in a randomized complete block design with 10 replications in 2014. Individual plot size was 0.6 m by 0.6 m and spacing between plots was 1 m. Seeds were coated with Laponite RD® gel of a rate of 0.012 g ml^−1^ of water (Colquhoun et al., 2001) to prevent displacement of the shallowly-sown kochia seeds by wind and to create a moist environment for seed germination. A 12 ml syringe was used to place 10 ml of solution containing approximately 10 kochia seeds on the soil surface. After kochia germination, seedlings were thinned to the respective proportions of target plant to neighbor densities. For the 10 and 35 neighbor plants m^−2^ treatment, the distance from target to neighbor was 15 cm while for the 70 neighbors m^−2^ treatment, the distance from target to the immediate neighbor and between neighbors was about 10 cm.

In 2015, five kochia populations were used as targets excluding the WA-S population to allow efficient management of plots because of additional treatments. Four kochia neighbor density treatments were evaluated by adding a no neighbor treatment. A complementary study in 2015 included corn as a neighbor at densities of 0, 10, and 35 corn plants m^−2^ to evaluate effect of crop density on the target plants. Two treatment factors in the kochia-only study were five kochia populations and four kochia neighbor densities while in the corn-neighbor study, two treatments factors were five kochia populations and three corn densities. Both studies were conducted within the same randomized complete block design with seven replications. Individual plot size was 0.6 m by 0.6 m and spacing between plots was 3 m. In 2015, seedlings were grown in the greenhouse and transplanted into the experiment rather than direct seeding. In the greenhouse, kochia seeds were shallowly sown in 25 by 15 by 2.5-cm plastic trays filled with commercial potting mixture (Miracle Gro, Marysville, OH). The trays were placed in a no-hole runoff catchment tray and water was added to the tray for sub-irrigation. The greenhouse was maintained at 25/20 C day/night, 60% relative humidity, and 15/9 h day/night photoperiod, supplemented with 120 μmol m^−2^ s^−1^ illumination provided by sodium vapor lamps. Kochia seedlings of about 5 cm tall were transplanted into the field in their respective target plot. In the case of corn as neighbors, corn seeds were directly sown in the field and the respective kochia seedlings (about 5 cm tall) as target plants were transplanted at corn emergence. For the 10 and 35 plants m^−2^, the target to neighbor distant was about 15 cm while for the 70 plants m^−2^, the distance from target to the immediate neighbor and between neighbors was about 10 cm.

The neighbor densities at the commencement and end of each trial were recorded, and the neighbor biomass was collected at the end of the season. Kochia target plant height, stem diameter at base of the plant, and plant canopy width (as a measure of the widest canopy) were collected biweekly starting from 3 weeks after planting in 2014 or at transplanting in 2015. Days to first flowering were recorded, from sowing in field or greenhouse, as a phenological variable. Target plants were harvested at 130 and 120 days after establishment in the field in 2014 and 2015, respectively. Fresh weight of the harvested target plants were recorded, plants placed in oven at 40 C for about 72 h and dry weights recorded. Seeds were collected by stripping them off the plants and cleaned using an air column separator. Total and 1000-seed weight per plant were measured. Total seed number (TS) was calculated for each plant:

(2) $$\begin{array}{l} \text{TS}=({\text{SW}}_{\text{T}}/{\text{SW}}_{1000})\times 1000 \end{array}$$

where SW_T_ is total seed weight (g plant^−1^) and SW_1000_ is weight of 1000 kochia seed (g). Growing degree days (GDD) for each day after sowing of seeds were calculated as recommended by Schwinghamer and Van Acker (2008):

(3) $$\begin{array}{l} {\text{GDD}}_{\text{daily}}=[({\text{T}}_{\text{max}}+{\text{T}}_{\text{min}})/2]-{\text{T}}_{\text{base}} \end{array}$$

(4) $$\text{Cumulative GDD}=\sum_{\text{i}=1}^{\text{n}}\text{GDDdaily}$$

where T_max_ is the maximum daily air temperature, T_min_ is the minimum daily air temperature, and T_base_ is the base temperature at which plant growth and development was deemed not to occur (0 C); n is the number of days elapsed from sowing date, and GDD_daily_ was a nonnegative value (daily GDD values that were negative were replaced by 0). A base temperature of 0 C seemed reasonable given that kochia has been known to emerge early in the spring (Dille et al., 2017) and 0 C has been used previously as a biologically justifiable base temperature for modeling the germination and emergence of kochia (Schwinghamer and Van Acker, 2008).

Growth of kochia populations over time was modeled using plant height as a function of cumulative GDD with the following three-parameter sigmoid function:

(5) $$\begin{array}{l} \text{H}=\text{a}/[\text{1}+\text{exp}(-(\text{x}-\text{c})/\text{b})] \end{array}$$

where H is the target plant height (cm) at cumulative GDD (x), while parameter *a* is the final height (cm), parameter *c* is the cumulative GDD required to attain 50% of the maximum height, and parameter *b* is the slope of the curve at the inflection point (and near 50% cumulative GDD). The growth regression curve and analysis were done using SigmaPlot V.12.3 (Systat Software, Inc).

Due to changes in the neighbor density between the commencement and end of the field trial, analysis of covariance (ANCOVA) was used to analyze the effects of the factors (neighbor densities and populations) on the fitness variables where density at the end of the season was considered as the random effect using the generalized linear mixed model GLIMMIX procedure of SAS 9.4 software (SAS Institute Inc., Cary, NC), which did not necessarily assume normal distribution of data, and was found suitable for the collected fitness data.

## Results

### Characterization of kochia populations to GR and GS: discriminatory glyphosate dose assay

Based on visual assessment of survival at 4 week after glyphosate application, all kochia plants from Scott (SC-R) and Thomas (TH-R) populations survived and 77% of kochia plants from Finney (FN-R) population survived the field use rate of glyphosate (Table 1). One out of 17 plants from the Wichita (WI-S) population survived while no plants survived from the Phillips (PH-S) and Wallace (WA-S) populations at the field use rate of glyphosate. The average injury score corresponded to the survival rating for each of these populations (Table 1). Thus, it was confirmed that SC-R, FN-R and TH-R populations were GR, while PH-S, WA-S, and WI-S populations were identified as GS in response to the field use rate of glyphosate. When treated with double the field rate of glyphosate, all kochia plants from the three GS populations did not survive, while 12, 30, and 13% of kochia plants from the SC-R, FN-R, and TH-R populations survived, respectively (Table 1). This suggests that the resistant populations were still segregating for glyphosate resistance.

**Table 1:** Survival (% of total individuals) and injury score at 4 wk after herbicide treatment of six kochia populations from western Kansas.

		**Populations**						
**Herbicide^b^**	**Mode of action**	**Rate (kg ha^−1^)**	**SC-R^a^**	**FN-R**	**TH-R**	**PH-S**	**WA-S**	**WI-S**
			**% Survival**					
Glyphosate^c^	EPSPS inhibitor	0.84 ae	100	77	100	0	0	7
		1.68 ae	12	30	13	0	0	0
			**Injury score**					
Glyphosate	EPSPS inhibitor	0.84 ae	1.1 B^d^	2.0 B	1.0 B	8.9 A	8.9 A	8.8 A
		1.68 ae	6.0 AB	6.6 AB	3.6 B	9.0 A	9.0 A	9.0 A

*Injury score was on the scale of 1–9, where 1 represents no injury and 9 represents completely injured (or dead). Total number of individuals in each population from two trials were 17*.

a *SC-R, FN-R and TH-R were suspected glyphosate resistant populations from Scott, Finney, and Thomas Counties, respectively, while PH-S, WA-S and WI-S were expected glyphosate susceptible populations from Phillips, Wallace and Wichita Counties, respectively in western Kansas*.

b *Herbicide treatments were applied to 8- to 10-cm tall kochia plants*.

c *with ammonium sulfate at 2% v/v*.

d *Injury score followed by the same letter within a row (among populations) are not significantly different based on Tukey's honestly significant difference at P < 0.05*.

### Characterization of kochia populations to GR and GS: shikimate accumulation assay

**Figure 2:**
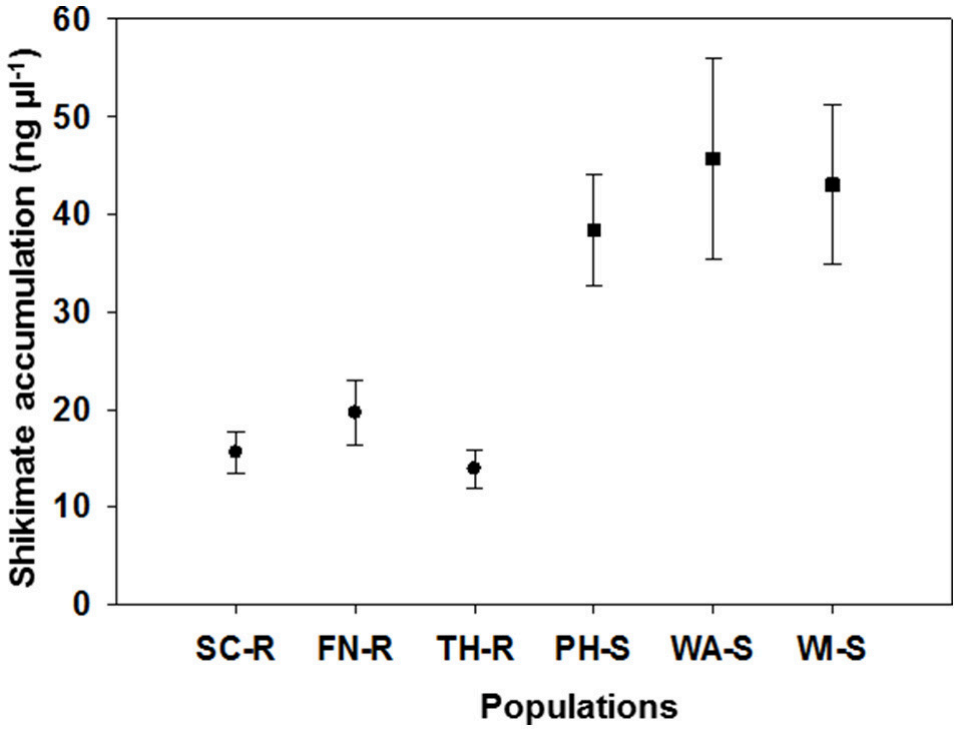
Mean (and standard error) for shikimate accumulation (ng ul^−1^) of 10 representative plants for each of six kochia populations. Suspected glyphosate-resistant populations were from Scott (SC-R), Finney (FN-R), and Thomas Counties (TH-R) while suspected glyphosate-susceptible populations were from Phillips (PH-S), Wallace (WA-S) and Wichita Counties (WI-S) in western Kansas.

The average shikimate accumulation in each GS population (43 ng μL^−1^) was significantly greater than for each of the GR populations (18 ng μL^−1^) as shown in Figure 2. Generally, at a discriminating dose of 100 μM, it is expected that there will be differential shikimate accumulation between GR and GS individuals (Shaner et al., 2005; Gaines et al., 2010). The use of shikimate accumulation had been previously used as a rapid nondestructive method for characterization of GR and GS kochia biotypes (Jugulam et al., 2014; Godar et al., 2015; Kumar et al., 2015). Overall, these results confirm that three of the kochia populations were truly GR including those from SC, FN, and TH counties, while the other three populations from PH, WI, and WA counties were GS. The mechanism of glyphosate resistance in the three GR populations was confirmed to be an increase in EPSPS gene copy (Osipitan, 2016).

### Germination characteristics

**Figure 3:**
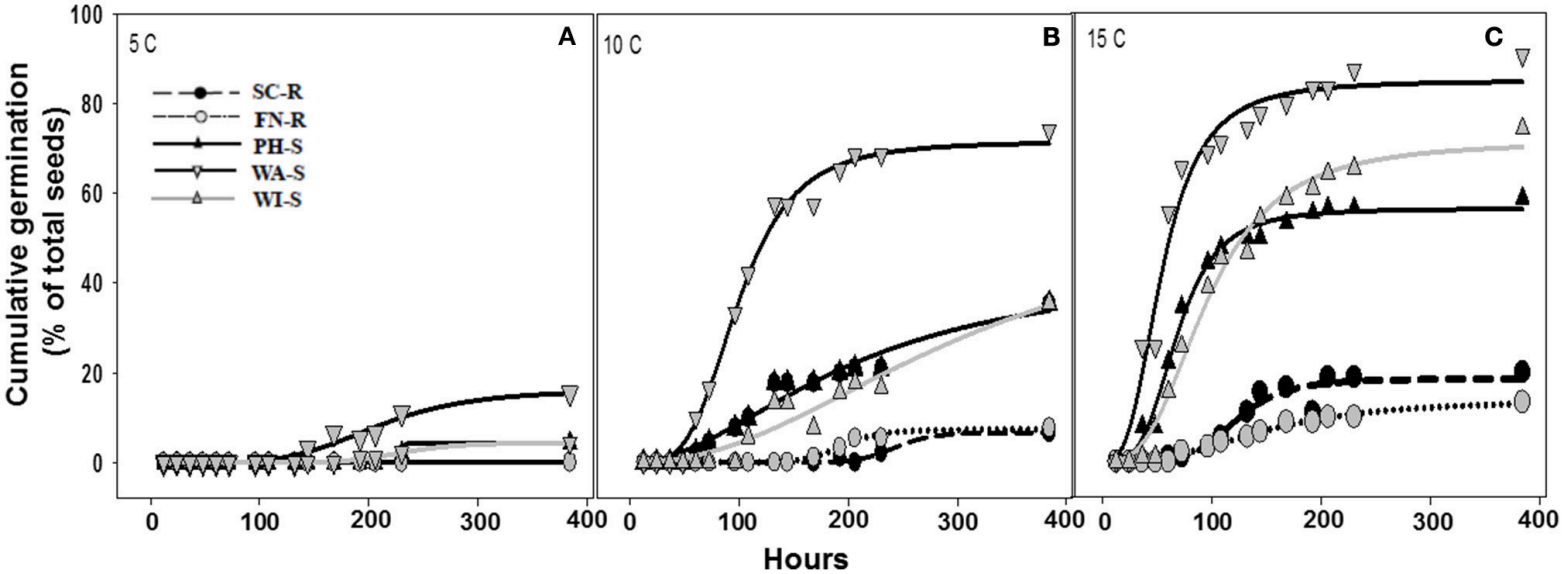
Cumulative germination as a percentage of 30 seed **(A–C)** at one of three constant temperatures of 5, 10, and 15 C. Germination dynamics of glyphosate-resistant and -susceptible kochia populations are modeled with Equation 1. Parameter estimates for each model are included in Table 2. Plots with solid lines and triangle symbols represent the GR populations while those with broken lines and circle symbols represents the GS populations.

The regression model (Equation 1) for each population fit the germination data as all parameter estimates were significantly different from zero (*P* < 0.001) on the basis of *t*-tests (data not shown) and coefficient of determination (R^2^) was sufficient (0.56–0.94) except when there was poor germination (Table 2). Maximum cumulative seed germination increased with increase in temperature from 5 to 15 C while time required to reach 50% of the maximum cumulative germination was shorter with increase in temperature for all populations (Figure 3, Table 2). None of the GR populations germinated at 5 C, while the GS populations had maximum cumulative germination ranging from 4 to 16% of total seed (Figure 3, Table 2). At 10 and 15 C, the two GR populations had greater maximum cumulative germination than the GS populations (Figure 3, Table 2). The maximum germination in respect to percent of total seeds for PH-S was the least (44% for 10 C and 56.3% for 15 C) compared to other GS populations. At low temperature (5 C), the time required to reach 50% of the maximum cumulative germination could not be estimated for the GR populations due to very low germination however, among the GS populations there was no difference in required time to attain 50% germination. The differences among the GR and GS populations were more obvious as temperature increased from 10 to 15 C (Table 2). At 15 C, the GR populations consistently required more time to reach maximum germination than the GS populations. Generally, viability test after final germination count, showed that GR populations had more nonviable seeds than the GS populations (data not shown).

**Table 2:** Parameter estimates obtained from logistic regression model (Equation 1) to describe the germination dynamics in % of total seed for each kochia population.

**Populations**	**Parameter estimates^b^**								
	**b**			**d**			**t**_**0**_		
	**5 C**	**10 C**	**15 C**	**5 C**	**10 C**	**15 C**	**5 C**	**10 C**	**15 C**
				**%**			**hours**		
SC-R^a^	*n*^c^	−14.1	−5.5	*n*	6.6 C^d^	18.4 C	*n*	243 A	122 A
FN-R	*n*	−10.6	−2.7	*n*	7.4 C	13.7 C	*n*	195 A	148 A
PH-S	−2.6	−1.9	−3.6	4.4	43.8 B	56.3 B	1092	200 A	68 C
WA-S	−5.3	−3.5	−2.9	16.1	71.4 A	84.8 A	214	99 B	54 D
WI-S	−2.4	−2.2	−2.9	4.2	57.5 A	71.2 A	976	313 A	95 B

a *SC-R and FN-R were glyphosate-resistant kochia populations from Scott and Finney Counties, respectively while PH-S, WA-S and WI-S were glyphosate-susceptible populations from Phillips, Wallace and Wichita Counties, respectively*.

b *Parameter estimates: b is the slope, d is the maximum % cumulative germination (of total seeds), and t_o_ is time (hours) required to reach 50% of maximum cumulative germination*.

c *No parameters could be estimated due to very poor germination*.

d *Within a column (across populations), parameter estimates with different letters were significantly different (P < 0.05), while parameter estimates with no letters had significant different differences among populations. Comparison of estimates among population was conducted using Tukey's honestly significant difference test (α = 5%)*.

### Vegetative growth and fecundity

#### Effect of neighbor density on target plant height over time

**Figure 4:**
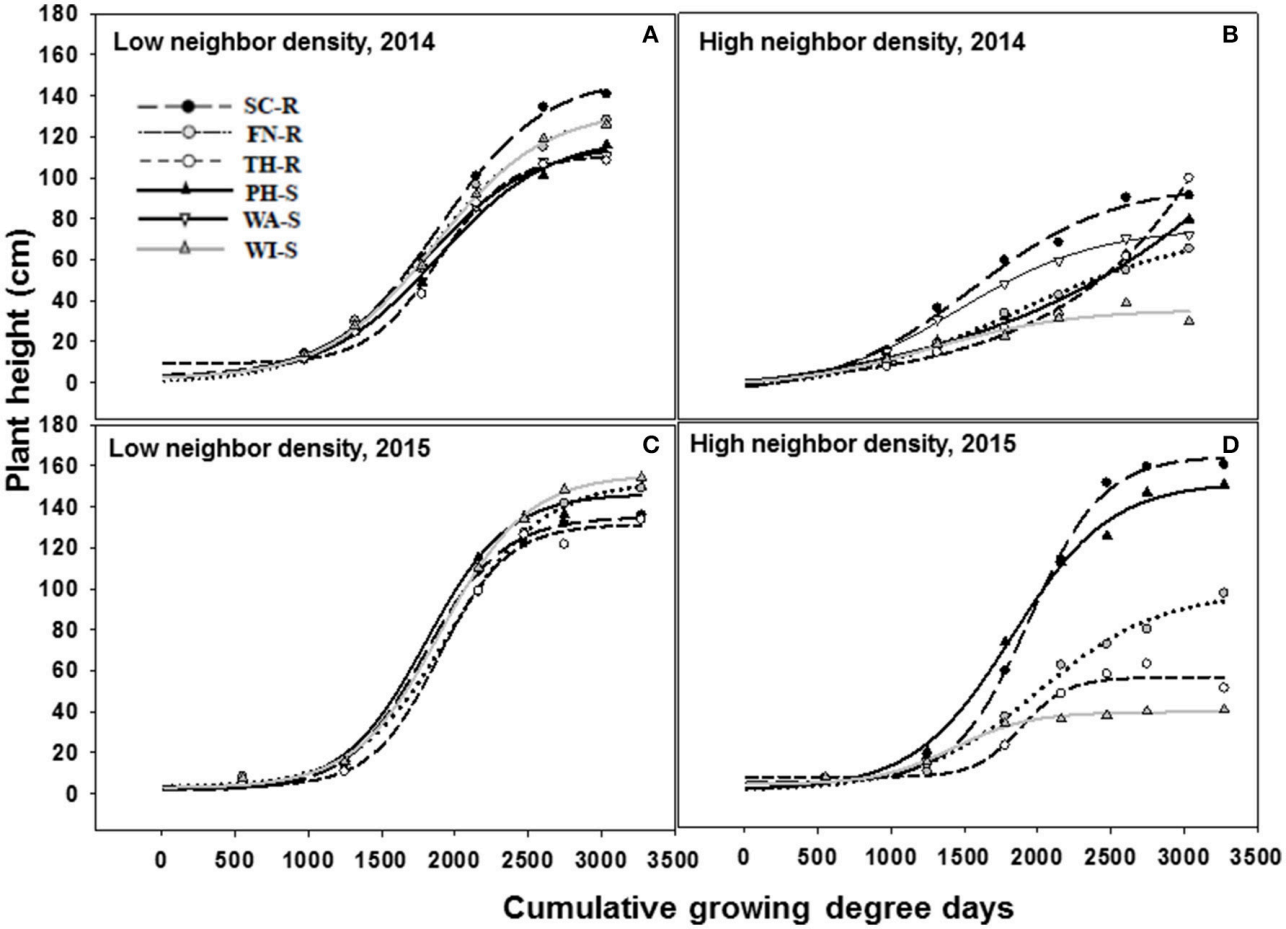
Increase in kochia plant height (cm) as a function of cumulative growing degree days (Tbase = 0 C, from the date of planting) at low (10 plants m^−2^) and high (70 plants m^−2^) kochia neighbor density using a three parameter sigmoid model (Equation 5) in 2014 and 2015. Resistant populations are represented by broken lines and mean values in circles while the susceptible populations are represented by solid lines and mean values in triangles. **(A–D)** represents low neighbor density for 2014, high neighbor density for 2014, low neighbor density for 2015, and high neighbor density for 2015 respectively. Tables 3, 4 shows the parameter estimates of the model.

The three-parameter sigmoid model (Equation 5) adequately fit the data for plant height over time. The rate of change in plant height as measured by the slope (b estimate) was not different among populations under the influence of low or high kochia neighbor densities in both 2014 and 2015 (Figure 4, Tables 3, 4). Similarly, there was no difference in the rate of change in stem diameter or canopy spread among populations over time (data not shown).

**Table 3:** Parameter estimates (±SE) and coefficient of determination (R^2^) for plant height of each kochia population and year in response to low neighbor density when Equation 5 was fit to data.

**Year**	**Population**	**Parameter estimates (±SE)**			**R^2^**
		**a**	**b**	**c**	
		**cm**		**CGDD**	
2014	SC-R	146 (21)	360 (112)	1,894 (108)	0.80
	FN-R	133 (36)	381 (220)	1,841 (223)	0.61
	TH-R	102 (15)	242 (106)	1,891 (107)	0.67
	PH-S	118 (21)	381 (138)	1,881 (133)	0.73
	WA-S	115 (26)	371 (176)	1,779 (180)	0.58
	WI-S	131 (30)	373 (186)	1,864 (183)	0.63
	*P*-value	0.038	NS	NS	
2015	SC-R	133 (11)	268 (79)	1,793 (84)	0.89
	FN-R	149 (26)	323 (146)	1,961 (155)	0.68
	TH-R	128 (10)	233 (67)	1,893 (72)	0.84
	PH-S	144 (12)	278 (72)	1,802 (77)	0.85
	WI-S	153 (8)	298 (46)	1,904 (49)	0.94
	*P*-value	NS	NS	NS	

*Parameter a represents plant height at harvest, b represents the slope of the curve, and c represents the cumulative growing degree days at 50% of the maximum height*.

*CGDD, Cumulative growing degree days*.

*NS, Not significant (P > 0.05)*.

*Estimates with different letters are significantly different (α = 0.05) within a column*.

**Table 4:** Parameter estimates (SE) and coefficient of determination (R^2^) for plant height of each population and year in response to high neighbor density when Equation 5 was fit to data.

**Year**	**Population**	**Parameter estimates (±SE)**			**R^2^**
		**a**	**b**	**c**	
		**cm**		**CGDD**	
2014	SC-R	102 (28)	4817 (244)	1545 (244)	0.59
	FN-R	80 (77)	634 (902)	1866 (826)	0.29
	TH-R	97 (24)	927 (213)	1276 (787)	0.48
	PH-S	99 (28)	1576 (662)	1629 (600)	0.45
	WA-S	79 (38)	469 (417)	1466 (425)	0.34
	WI-S	38 (13)	483 (311)	1265 (294)	0.53
	*P-*value	0.016	NS	NS	
2015	SC-R	159 (17)	251 (81)	1944 (89)	0.82
	FN-R	96 (26)	411 (235)	2006 (247)	0.63
	TH-R	48 (14)	158 (198)	1889 (227)	0.24
	PH-S	149 (21)	335 (121)	1827 (128)	0.74
	WI-S	35 (13)	260 (313)	1422 (366)	0.31
	*P*-value	<0.001	NS	NS	

*Parameter a represents plant height at harvest, b represents the slope of the curve and c represents the cumulative growing degree days at 50% of the maximum height*.

*CGDD, Cumulative growing degree days*.

*NS, Not significant (P > 0.05)*.

*Estimates within a column with different letters are significantly different (α = 0.05)*.

At low neighbor density, the estimated final plant height (parameter “a”) was different among populations in 2014 (Figure 4A, Table 3) but not different in 2015 (Figure 4C, Table 3). At high neighbor density in both years, there were differences in estimated final plant height among populations (Figures 4B,D, Table 4), however, ANCOVA showed that there was an interaction between populations and densities for the observed final plant height (Table 5).

**Table 5:** *P*-value of analysis of covariance for fitness variables of populations at varying kochia neighbor densities.

**Fitness variables**	**Population**		**Density**		**Population by density**	
	**2014**	**2015**	**2014**	**2015**	**2014**	**2015**
Biomass at harvest (g)	NS	0.025	0.001	0.001	0.012	0.003
Stem diameter at harvest (cm)	NS	NS	NS	NS	NS	NS
Plant width at harvest (cm)	–	NS	–	0.001	–	NS
Days to flowering	0.004	0.017	0.032	NS	NS	NS
Total seed weight (g plant^−1^)	NS	0.048	0.001	0.001	NS	0.013
1,000 seed weight (g)	NS	NS	NS	NS	NS	NS
Total seed number (plant^−1^)	NS	0.001	0.001	0.001	NS	0.016
Reproductive Effort	NS	NS	NS	NS	NS	NS

*NS, Not significant at p > 0.05*.

The recorded differences in final plant height were not consistent among GR and GS populations. For instance, in competition with low neighbor density, TH-R was the shortest among populations at the end of the season and was about 68% of the tallest recorded population (SC-R) in 2014 (Figure 4A, Table 3). While in competition with high neighbor density averaged over years, TH-R and WI-S plants were about 55 and 30% of the average height attained by SC-R (130 cm), and 58 and 29% of the average height attained by PH-S (124 cm), while three populations (SC-R, PH-S, and FN-R) had similar heights at harvest (Figures 4B,D, Table 4).

Phenology estimate in the model showed that thermal time, cumulative GDD required to attain 50% of maximum height, was not different among populations and the cumulative GDD of each population fall between 1,265 to 1,894 GDD in 2014 and 1,422 to 2006 GDD in 2015 across neighbor densities (Tables 3, 4). Going by these estimates, there was no evidence that temperature required for increase in height differed among the populations.

#### Effect of neighbor density on target plant biomass, days to flowering, and fecundity

Analysis of covariance of growth and reproductive measurements at the end of season showed that there were interactions between the two main factors of kochia population and kochia neighbor density for target plant biomass in both years, as well as for seed weight and number of seeds produced per plant in 2015 (Table 5). Across populations, as neighbor density increased, plant biomass decreased by 80% in 2014 and 85% in 2015 (Table 6) while total seed weight and number per plant were reduced by 71 and 80%, respectively (Table 7) in 2015. The differences among populations were significant at high neighbor density for these measurements (Tables 7, 8), however, the differences among populations were not in respect to glyphosate resistance.

**Table 6:** Means of target kochia biomass (g plant^−1^) at different levels of kochia neighbor densities.

**Year**	**Population**	**Kochia neighbor densities**			
		**Low**	**Moderate**	**High**	***P*-value**
		**g plant^−1^**			
2014	SC-R	1556	86	87 a	0.002
	FN-R	1430	389	127 a	0.01
	TH-R	561	161	211 a	NS
	PH-S	1077	561	71 ab	NS
	WA-S	1118	254	318 a	NS
	WI-S	2302	57	17 b	0.001
	*P*-value	NS	NS	0.04	
2015	SC-R	315	181	66 a	NS
	FN-R	577	121	39 b	0.001
	TH-R	241	218	33 b	0.001
	PH-S	354	162	46 ab	0.04
	WI-S	477	141	19 c	0.001
	*P*-value	NS	NS	0.001	

*NS, Not significant*.

*Estimates with different letters are significantly different (α = 0.05) within a column*.

**Table 7:** Means of seed weight (g plant^−1^) and seed number (# plant^−1^) at different levels of kochia neighbor densities in 2015.

	**Seed weight**				**Seed number**			
	**Kochia neighbor densities**				**Kochia neighbor densities**			
**Population**	**Low**	**Moderate**	**High**	***P*-value**	**Low**	**Moderate**	**High**	***P*-value**
SC-R	63.50	37.40	13.1 a	0.16	146,112	47,663	16,074 a	0.02
FN-R	120.57	50.72	1.7 b	0.001	199,970	74,123	3,037 b	0.001
TH-R	65.31	65.32	1.32 b	0.001	41,086	62,626	1,105 b	0.001
PH-S	82.50	34.51	14.8 a	0.05	113,074	70,713	16,323 a	0.01
WI-S	74.60	39.01	0.82 b	0.001	80,591	22,358	1,294 b	0.001
*P*-value	NS	NS	0.001		NS	NS	0.001	

*NS, Not significant*.

*Estimates with different letters are significantly different (α = 0.05) within a column*.

**Table 8:** Analysis of covariance for target response under intra-specific (kochia) and interspecific (corn) neighbors for 10 and 35 plants m^−2^ neighbor densities.

**Fitness variables**	**Type of neighbor**			**Type of neighbor by density**
	**Corn**	**Kochia**	***P*-value**	***P*-value**
Plant height at harvest (cm)	128	143	0.02^*^	NS
Biomass at harvest (g plant^−1^)	239	292	0.04^*^	NS
Seed weight (g plant^−1^)	29.9	63.9	0.02^*^	NS
Seed number (# plant^−1^)	45,300	85,703	0.006^*^	NS

* *Significant difference at α ≤ 0.05*.

*NS, Not significant*.

Target plant biomass at harvest showed no differences among populations at low neighbor density but there were differences at high neighbor density; differences were not consistent among GR and GS populations (Table 6). Similarly, the effects of kochia neighbor density on total seed weight and seed number per plant were significant at high density where SC-R and PH-S had similar total seed weights (13.1–14.8 g plant^−1^) and these were greater than the seed weight of FN-R, TH-R, and WI-S (Table 7). The impact of high neighbor density compared to low neighbor density on plant biomass and seed production among populations might have been exaggerated. At low density, the proximity of target to neighbor was 15 cm while at high density it was only 10 cm, hence there was confounding effect of target to neighbor distance and number of neighbors at each density level. However, the evaluation of proximity effect may not be necessary for this study, as the crowding treatment for all populations was similar and this study was more concerned about the response of each population to the crowding level.

The type of neighbor (kochia or corn) had an effect on target plant height, biomass, seed weight, and seed number (Table 8), such that corn suppressed target kochia plant variables more than kochia as a neighbor. The impact of type of neighbor on the populations was not influenced by the density of neighbor (Table 8). The ANCOVA of pooled populations for GR or GS showed that there was no difference for plant height, stem diameter, plant width, days to flowering, seed weight, 1000-seed weight, number of seed per plant, or reproductive effort based on resistance (Table 9).

**Table 9:** *P*-values of the analysis of covariance for pooled populations [Resistant (R) vs. Susceptible (S)], neighbor density and interaction.

**Fitness variables**	**R vs. S**		**Density**		**R vs. S by density**	
	**2014**	**2015**	**2014**	**2015**	**2014**	**2015**
Plant height at harvest (cm)	NS	NS	0.001^**^	0.001^**^	0.02^*^	NS
Biomass at harvest (g plant^−1^)	NS	NS	0.001^**^	0.001^**^	NS	NS
Stem diameter at harvest (cm)	NS	NS	0.001^**^	NS	NS	NS
Days to flowering	NS	NS	0.03^*^	NS	NS	NS
Total seed weight (g plant^−1^)	NS	NS	0.001^**^	0.001^**^	NS	NS
1,000 seed weight (g)	NS	NS	NS	NS	NS	NS
Total seed number	NS	NS	0.001^**^	0.001^**^	NS	NS
Reproductive effort	NS	NS	NS	NS	NS	NS

* *Significant (≤ 0.05)*.

** *Highly significant (≤ 0.01)*.

## Discussion

The influence of temperature on speed and level of germination of GR and GS kochia populations is important in agronomy, as it relates to cohort establishment (periodic emergence of plants of the same species per unit land area in a growing season) and optimum time of weed control. Because GR populations had delayed germination at constant low temperatures, an early season weed management strategy with no effective supplemental weed control for subsequent cohorts will likely increase the proportion of GR individuals within a kochia population over time. Also, it is proposed that in this case, mixed populations GS individuals would have competitive advantage for limited resources through earlier seedling emergence with subsequent early canopy cover and root growth, reducing the growth and potential seed production of GR individuals in the absence of glyphosate applications.

The GR populations not only have delayed germination but also possess reduced ability to germinate at constant temperatures. In this study, the difference in total germination among GR and GS populations during the period of observation may be attributed to either differential seed viability or dormancy. Differences in seed viability are more likely as GR and GS populations had differential total germination even under ideal temperatures (10 C). Noting that this study was conducted 3 years after the seeds were collected suggests that seeds from GR populations may lose viability sooner than GS populations. Consequently, in a segregating population or a seed mixture of GR and GS kochia individuals, the frequency of the GR to GS may decrease over time in the absence of glyphosate application. Wakelin and Preston (2006) reported that at the end of 3 years, there was an observable decrease in proportion of surviving GR individuals in seeds of a segregating population of ryegrass (*Lolium rigidum*) from a cross between GR and GS parents. Most recently, van Etten et al. (2016) reported a negative correlation between GR and seed quality or seed germination in a study that examined 43 naturally-occurring populations of tall morningglory (*Ipomoea purpurea*) that varied in their level of GR. This was similar to an earlier report by Debban et al. (2015) that GR lines of tall morningglory had fewer viable seeds than GS lines.

The underlying factor contributing to differences in germination characteristics of GR and GS populations is yet to be understood. The GR kochia populations were known to have high EPSPS gene copies (Osipitan, 2016) and EPSPS gene copies in kochia were generally known to be highly functional for production of EPSPS enzymes (Godar, 2014; Kumar et al., 2015; Gaines et al., 2016). However, the reduced longevity or viability of GR seeds may not be unconnected with the postulation that metabolic energy cost associated with overproduction of EPSPS protein can be detrimental to other protein production since protein production or repair is known to be important to long term seed viability, particularly when seed are stored in dry state (Shen-Miller, 2002).

Any weed management strategy that is timely to remove both early and later emerging kochia seedlings, as well as a strategy that delays the germination of seeds to another growing season, can help reduce the frequency of GR individuals within a population on the field. One such strategy may include a residual soil-applied herbicide that would remove earlier germinating GS seedlings, and continue to be present for later germinating GR seedlings. Another strategy is the use of tillage to bury kochia seed to a soil depth that impedes seedling establishment (Schwinghamer and Van Acker, 2008) such that even when seed are repositioned (through tillage) to soil depth conducive for germination in a subsequent cropping season, many of the GR seeds could have lost their viability.

It was expected that overproduction of EPSPS enzyme that endowed GR in kochia and the consequent metabolic cost of production of this enzyme at the expense of other protein production needed for plant growth and fecundity will be a trade-off for plant fitness and this trade-off was expected to be more obvious under competition with neighbors for resources. However, in this field study, reduced growth rate and less fecundity were not observed, even under competition. This is similar to a previously reported greenhouse study by Kumar and Jha (2015). Different study environments (field and greenhouse) have now demonstrated that GR in kochia endowed by overproduction of EPSPS appears to have no associated cost with growth and seed production.

Differences among kochia populations in response to varying levels of neighbor competition were not clearly related to glyphosate resistance, but rather showed some consistency in direction of inherent ability of the populations to capture resources in the presence of neighbors. One GR population (TH-R) and one GS population (WI-S) consistently showed less vegetative growth and seed production at high neighbor density while there was no difference in growth and seed production of other populations (both GR and GS). If just two populations, such as SC-R vs. WI-S or TH-R vs. PH-S were compared for plant height, biomass accumulation or seed production, the differences could have been erroneously attributed to evolution of GR in the populations. The results suggest the importance of evaluating more than just two populations for fitness comparison, to check for consistency and allow validity. It is obvious that factor(s) other than GR trait resulted in differences of final plant height, biomass accumulation, and seed production among these populations. These differences could be as a result of their diverse genetic background (Mengistu and Messersmith, 2002; Giacomini et al., 2014) or adaptive features that evolved due to their original environmental conditions (Jasieniuk et al., 1996).

Use of pure-lines or isogenic lines collected from inbreeding of several filial generations has been recommended and has been previously used as reliable plant material for fitness studies (Vila-Aiub et al., 2009b; Giacomini et al., 2014; Kumar and Jha, 2015). Many studies used a single pair comparison between resistant and susceptible individuals of such lines to arrive at conclusion on fitness studies. This study did not evaluate such inbred lines. Kochia, as an obligate outcrossing species, demonstrates significant inbreeding depression, which may also mask fitness differences. Another approach may be to conduct comparisons among several pairs of resistant and susceptible inbred lines developed from different populations.

## Conclusions

Fitness costs as a result of evolution of herbicide resistance in plants cannot be generalized. Differences in methodology and interpretation of studies that attempt to quantify fitness costs or evolutionary trade-offs associated with evolved resistance to herbicide are very diverse. It is also very likely that identification of fitness costs will vary based on weed species, the mechanism of resistance involved, and the genetic background through which resistance is expressed. Nevertheless, conducting these studies to identify differences in life history traits had shown a great potential in detecting fitness cost.

Of the life history stages measured, fitness difference between the GR and GS kochia populations was consistently found in their germination characteristics. The GR populations showed less seed longevity, slower germination rate, and less total germination than the GS populations. Plant growth, days to flowering, and seed production were not necessarily different among GR and GS populations. In general, this study suggests that any weed management strategy that delays the germination and emergence of seeds can help reduce the frequency of GR individuals in a kochia population in the field. Otherwise, once the GR individuals germinate and become seedlings, they grow, and reproduce even under intense competition with either kochia ‘or’ crop neighbors. Thus, agronomic practices that would take advantage of differences in the seed biology of GR and GS should be explored to help minimize or reverse the evolution of GR in kochia populations.

## Author contributions

JD secured the funding and was major advisor for the graduate PhD degree of OO, the first author. JD initiated the study, supervised the study, provided laboratory and field materials needed for the study, review and edited manuscript drafts. OO modified the study design, conducted the study, analyzed and interpreted the data, and drafted the manuscript.

### Conflict of interest statement

The authors declare that the research was conducted in the absence of any commercial or financial relationships that could be construed as a potential conflict of interest.
